# Follicular guidance for oocyte developmental competence

**DOI:** 10.21451/1984-3143-AR2018-0035

**Published:** 2018-08-03

**Authors:** Satoshi Sugimura, Dulama Richani, Robert B. Gilchrist

**Affiliations:** 1 Department of Biological Production, Tokyo University of Agriculture and Technology, Tokyo 183-8509, Japan; 2 Discipline of Obstetrics & Gynaecology, Fertility and Research Centre, School of Women's & Children's Health, University of New South Wales Sydney, 2052, Australia

**Keywords:** cumulus cells, oocyte, oocyte secreted factors.

## Abstract

The advancement of folliculogenesis is coincident with the sequential acquisition of oocyte developmental competence. In practical bovine/porcine ART, cumulus-oocyte complexes (COCs) aspirated from small antral follicles have low developmental competence relative to COCs from medium/large antral follicles, as evidenced by a poor capacity to support embryogenesis up to the blastocyst stage. This is in part because of incomplete differentiation of cumulus cells in small antral follicles, in particular under-developed functionality of EGF signalling. Gonadotrophins and oocyte-secreted paracrine factors cooperate to establish EGF receptor functionality in cumulus cells, which appears to be involved in the acquisition of oocyte developmental competence. Here we review the modification of follicular cumulus cells during antral folliculogenesis involved in oocyte developmental competence.

## Introduction

The developmental competence of an oocyte, referring to its capacity to support fertilization, preimplantation embryo and subsequent full-term development, is inherently linked to environmental cues it receives from the follicular somatic cell compartment (Gilchrist and Thompson, 2007). The follicular soma is regulated principally by gonadotropic cues (FSH and LH) during the course of folliculogenesis (Scaramuzzi *et al*., 2011).

Oocytes acquire increasing developmental competence sequentially with advancing folliculogenesis and as such oocyte and follicle development are inter-dependent processes (Thibault, 1977). Hence, as the obligatory driver of folliculogenesis, FSH is also the central driver of the acquisition of oocyte developmental competence, facilitating a myriad of follicular somatic cell functions that nurture the growth and development of the oocyte. During the terminal phases of folliculogenesis and oogenesis, FSH promotes expression of the LH and epidermal growth factor (EGF) receptors on granulosa cells (Erickson *et al*., 1979; El-Hayek *et al*., 2014). FSH also facilitates the bilateral communication between granulosa cells and between the oocyte and granulosa/cumulus cells (CCs) by promoting gap junctional communication (GJC; El-Hayek and Clarke, 2015), likely via cyclic adenosine monophosphate (cAMP)-phosphate kinase A (PKA) pathway activation. Oocyte-CC GJC is responsible for the transfer of small molecules such as cAMP, cyclic guanosine monophosphate (cGMP) and metabolites from the CCs to the oocyte (Sugimura *et al*., 2014; Li *et al*., 2016). In addition, vesicles and potentially RNA may traffic to the oocyte from the CC’s transzonal projections (Macaulay *et al*., 2014) which, together with GJC, regulate oocyte meiotic maturation and developmental competence (Sugimura *et al*., 2014; Li *et al*., 2016; Russell *et al*., 2016).

Key propagators of the ovulatory cascade within the follicle are the EGF-like peptides; amphiregulin (AREG), epiregulin, and betacellulin, which are produced by mural granulosa cells in response to LH, and signal via the EGF receptor (EGFR) that is expressed on mural granulosa and CCs (Park *et al*., 2004; Shimada *et al*., 2006; Hsieh *et al*., 2007). EGFR activation, in cooperation with the oocyte-secreted factors (OSFs), stimulates gene expression that enables cumulus expansion and ovulation. Two OSFs that facilitate this and are essential for ovulation and oocyte capture by the infundibulum are bone morphogenetic protein 15 (BMP15) and growth differentiation factor 9 (GDF9), which are structurally similar members of the transforming growth factor β family that can also form a heterodimer termed cumulin (Mottershead *et al*., 2015). Hence, the final steps of oocyte development prior to ovulation are dictated by endocrine cues mediated by the gonadotrophins and by paracrine cues from the oocyte, intersecting in the CCs as the key facilitators of oocyte developmental competence (Russell *et al*., 2016; Richani and Gilchrist, 2018).

This review will focus on the recent advances in our knowledge of the contribution of endocrine and paracrine cues in the differentiation of follicular granulosa and CCs during antral folliculogenesis, and their contribution to oocyte developmental competence. This knowledge is important for the application of advanced reproductive technologies in domestic animal breeding and in humans. The successful clinical application of the key reproductive technologies of superovulation (combined with artificial insemination or IVF) and oocyte *in vitro* maturation (IVM) are critically dependent on this knowledge.

## EGFR signaling and oocyte developmental competence

It is now well established that the EGF network is an essential propagator of the ovulatory signal in the Graafian follicle to the cumulus-oocyte complex (COC; Park et al., 2004; Conti et al., 2012). The concept that the oocyte sequentially acquires developmental competence to support fetal development throughout antral follicle development has long been clear (Eppig *et al*., 1992; Lonergan *et al*., 1994). Hence, oocytes from small antral follicles have a significantly poorer capacity for cumulus expansion and for the support of early embryogenesis, compared with those from large antral follicles. Indeed this concept serves as the basis for the need for superovulation of women undergoing IVF and of cattle undergoing multiple ovulation and embryo transfer (MOET).

Prochazka *et al*. (2003) hypothesized that porcine COCs from small antral follicles (3-4 mm) have an under-developed EGFR. Indeed, there is a growing body of evidence showing that the development of a functional EGF network in follicular granulosa cells is suppressed until the peri-ovular stage as a mechanism of limiting ovulation to the dominant follicle(s), and thus to the developmentally competent oocyte(s); Richani and Gilchrist, 2018). The exact timing of acquisition of functional EGFR signalling in COCs and the associated molecular changes in the oocyte are still unclear, but for experimental purposes such studies commonly divide the antral phase of folliculogenesis in half into small versus large antral follicles. In pigs for example, <4mm (small follicles) vs. >4 mm (medium-large follicles) are typically compared (Prochazka *et al*., 2003; Ritter *et al*., 2015), whereas in cattle which ovulate larger follicles, a medium-large antral follicle is 6-10 mm (Lonergan *et al*., 1994). Using such models, porcine COCs derived from small antral follicles (<4 mm) are unresponsive to all EGF family ligands and COCs exhibit progressive acquisition of EGF responsiveness with follicle growth (Prochazka *et al*., 2000, 2003; Diaz *et al*., 2007; Ritter *et al*., 2015). The acquisition of EGF signalling capability by CCs with advancing follicle growth coincides with oocyte acquisition of developmental competence, and the two are likely to be related (Ritter *et al*., 2015; Sugimura *et al*., 2015). These latter recent findings suggest that the EGF network may contribute to oocyte quality by providing regulatory cues from the cumulus cells to the oocyte which regulate oocyte integrity (Gilchrist and Richani, 2013; Richani and Gilchrist, 2018). The EGF network also mediates mRNA translation in the transcriptionally silent oocyte during the course of meiotic maturation to the metaphase II stage (Chen *et al*., 2013). This directly impacts oocyte quality as genetic perturbation of *Areg* expression in mouse cumulus cells leads to reduced fecundity (Chen *et al*., 2013).

**Figure 1:**
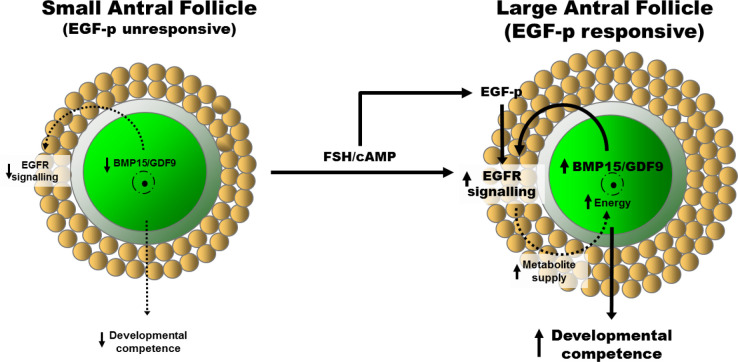
Hypothetical mechanism of cooperation between endocrine FSH priming and oocyte paracrine signals to promote EGFR signaling in cumulus cells. The acquisition of EGFR signaling capability by cumulus cells is a developmental hallmark for the COC. Cumulus-oocyte complexes derived from small antral follicles, which have low developmental competence, exhibit under-developed EGFR signaling as they are unresponsive to EGF-peptides (EGF-p). As folliculogenesis progresses, EGFR functionality is induced in COCs by the concerted actions of FSH/cAMP and oocyte-secreted factors (of which BMP15 and GDF9/cumulin have been identified), and is associated with increased oocyte developmental competence. Improved oocyte developmental competence may be facilitated by EGF-peptide stimulation of cumulus cell glycolysis and provision of metabolites (e.g. NAD(P)H) to the oocyte facilitating oocyte mitochondrial activity and energy production needed for development. Figure from Richani and Gilchrist (2018) adapted from Sugimura *et al*. (2015) with permission.

An understanding of the cellular and molecular mechanisms leading to the development of EGF signalling capabilities with follicle development can provide new approaches for improving the success of oocyte IVM, a reproductive technique that involves the maturation of COCs derived from small antral follicles of unstimulated ovaries (Gilchrist, 2011). Granulosa-type cells (preantral, mural, and cumulus) express EGFR mRNA and protein throughout folliculogenesis, and is activated by a range of EGF family members to promote follicle growth (Roy 1993; Garnett *et al*., 2002). Several studies have shown that EGFR mRNA expression is lower in small antral follicle CCs than their large antral follicle counterparts (Singh *et al*., 1995; Prochazka *et al*., 2003; Caixeta *et al*., 2009; El-Hayek *et al*., 2014). However, in a porcine model, we observed (Ritter *et al*., 2015) CCs from small antral follicles (<4 mm) exhibit equal expression of EGFR transcripts as those from larger antral follicles (>4 mm), however EGFR protein production and phosphorylation and subsequent downstream ERK1/2 activity were perturbed. Hence, COCs from small antral follicles are unresponsive to EGF peptides and cannot undergo expansion. There is now good evidence that the EGF signalling network is under-developed in COCs from small antral follicles (Richani and Gilchrist, 2018; Fig. 1).

## Establishing cumulus EGFR signaling requires orchestration by endocrine cues and oocyte-secreted factors

Details of the mechanisms responsible for the maturation of the EGF network in CCs remain unclear, however recent evidence suggests that cooperation between endocrine (FSH) signaling and paracrine signals from the oocyte is essential in this process. FSH plays a major and essential role in follicle development. Follicles of Fshb^-/-^ null mice exhibit perturbations in CC differentiation and oocyte meiotic maturation, as well as a deficiency in follicle Egfr mRNA expression (El-Hayek *et al*., 2014). FSH sensitivity is long understood to be a determining factor in follicle selection; recent evidence suggests that this may in part be due to FSH promoting development of a functional EGF network throughout antral follicle growth. Prochazka *et al*. (2003) demonstrated that *in vitro* treatment of porcine small antral COCs with FSH promotes EGF responsiveness and subsequent EGFR signaling. EGFR signaling is essential for cumulus expansion, and requires activity of the FSH downstream effector protein kinase A (PKA; Prochazka *et al*., 2012). Hence, evidence from mice and domestic animal models suggests that endocrine cues are needed for the maturation of the EGF signaling network in CCs, and that the FSH-cAMP-PKA signalling axis mediates this.

Recent evidence has shown that the oocyte itself also significantly contributes to the development of the EGF network throughout the antral phase of folliculogenesis by mediating the effects of FSH. Alone, FSH is insufficient in inducing EGF responsiveness in granulosa and cumulus cells (Fig. 1). Prior to differentiation into cumulus cells, preantral granulosa cells are unable to undergo expansion. Diaz *et al*. (2006, 2007) showed that this is due to the immaturity of the pre-antral oocyte; murine preantral granulosa acquire the capacity to undergo EGF-induced expansion if cultured with oocytes from large antral follicles as well as treated with FSH, suggesting that the oocyte modulates its secretome to regulate the follicle’s response to external endocrine cues. In addition, Sugimura *et al*. 2015) recently showed that small antral porcine COCs can be induced to respond to AREG following exposure to the FSH effector cAMP. However, this required the exogenous addition of GDF9 or BMP15, since cAMP alone had little effect on AREG-induced meiotic maturation, cumulus expansion, ERK1/2 phosphorylation, or blastocyst development in small antral oocytes in the absence of GDF9 and BMP15 (Sugimura *et al*., 2015). This is consistent with the findings that GDF9 and BMP15 facilitate CC EGFR expression via SMAD2/3 (Su *et al*., 2010), and that for full GDF9 signalling to occur, cooperation between the EGFR-ERK1/2 and SMAD2/3 pathways in granulosa and cumulus cells is required (Sasseville *et al*., 2010). EGF responsiveness can be induced in CCs from small antral follicles (<4 mm) by co-culture with oocytes from larger antral follicles (>4 mm) (Ritter *et al*., 2015). In contrast, EGF responsiveness in CCs from small antral follicles cannot be induced by co-culturing with low competence oocytes of small antral follicles, demonstrating that native factors secreted from developmentally competent oocytes mediate this process, and that the oocyte alters its secretome throughout folliculogenesis to regulate CC acquisition of EGFR signaling. This suggests that EGF responsiveness is a milestone in the growth and development of the COC throughout folliculogenesis (Ritter *et al*., 2015). This mechanism is likely in place to perturb meiotic resumption and ovulation of oocytes that have not completed folliculogenesis which are growing in the presence of continual surges of endocrine LH and FSH during menstrual cycles. Hence, in the same way that FSH promotion of LH receptor signalling marks the development of mural granulosa cells, dual oocyte- and endocrine FSH-induction of EGF responsiveness in CCs represents a developmental milestone in folliculogenesis (Fig. 1; Ritter *et al*., 2015; Richani and Gilchrist, 2018).

## Modification of cumulus cells by FSH-priming

As mentioned above, promotion of EGFR signalling in CCs might be a key component in the acquisition of oocyte developmental competence (Ritter *et al*., 2015; Richani and Gilchrist, 2018). However, this is not the full story since the CCs of mouse COCs derived from small antral follicles which have received no gonadotropin priming are able to undergo a degree of expansion *in vitro* (Vanderhyden *et al*., 1990; Wigglesworth *et al*., 2015). CCs from small antral follicles exhibit a more immature capacity for metabolism, inter-cell communication and cell differentiation at the transcriptome level (Wigglesworth *et al*., 2015), suggesting that the EGFR network is likely one of several signaling pathways which develop with follicle growth to participate in the acquisition of oocyte developmental competence.

**Figure 2:**
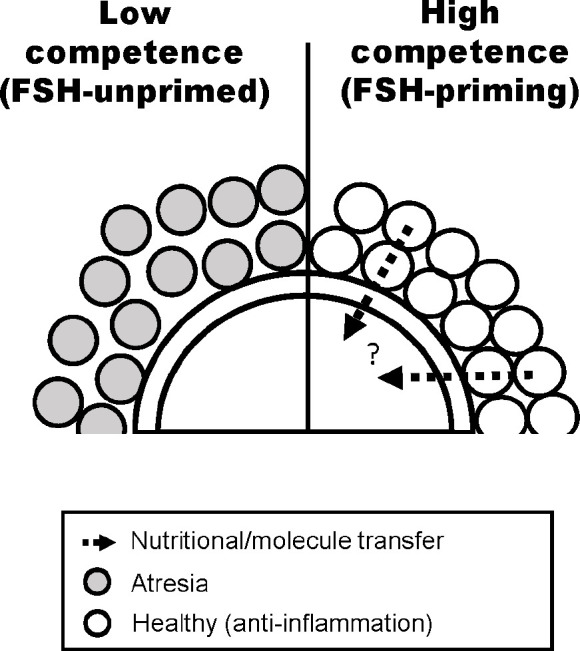
Hypothetical model of the effect of FSH-priming on bovine cumulus cells based on RNA-seq expression signature. Cumulus cells in a low competence model without FSH-priming exhibit a transcriptomic signature suggestive of decreased inter-cell communication, increased atresia, and driving a spontaneous ovulation-like cascade. Conversely, cumulus cells in a high competence model following FSH-priming are in a state of increased inter-cell communication which promotes the transport of molecules from cumulus cells to oocytes, and exhibit increased anti-inflammatory signals at the time of final oocyte maturation. Figure adapted from Sugimura *et al*. (2017).

*In vivo* priming of follicles prior to final oocyte maturation enhances oocyte developmental competence (Hendriksen *et al*., 2000). This priming can be driven by exogenous FSH administration; the oocytes derived from cows subjected to FSH treatment prior to oocyte collection are more developmentally advanced than those from unstimulated cows (Sirard *et al*., 1999; Sugimura *et al*., 2012), likely due in large part to the drastically altered transcriptome it induces in CCs (Fig. 2; Sugimura *et al*., 2017). Our recent RNA-seq analysis showed substantial alteration to CC gene transcription following *in vivo* FSH-priming, with the majority of altered transcriptomic genes being downregulated (Sugimura *et al*., 2017). Surprisingly, the matrix-formation genes HAS2, TNFIP6, and PTX3, which are typically up-regulated in CCs during oocyte maturation and are associated with oocyte developmental capacity (Gebhardt *et al*., 2011; Wathlet *et al*., 2011), were downregulated, however this may be attributed to the timing of cell collection, whereby CCs were collected from immature unexpanded COCs (Sugimura *et al*., 2017).

FSH-priming also enhances CC transcripts associated with interferon signaling and interferon regulatory factor (IRF) activation, including interferon-stimulated genes (Sugimura *et al*., 2017). IRF7, a regulator of the viral IFNα/β immune response (Honda *et al*., 2005), ranked first in the list of activated upstream regulators in CCs from FSH-primed cows (Sugimura *et al*., 2017). TGFB1 regulates IRF7 expression whereby prolonged exposure to TGFB1 promotes downregulation of IRF7 (Cohen *et al*., 2014). In granulosa cells, increased TGFB1 is a hallmark of the activated inflammatory process, which may stimulate follicular atresia (Hatzirodos *et al*., 2014a). Pathway analysis predicted TGFB1 as an inhibited upstream regulator and the expression of TGFB1 and the receptor TGFR2 were lower in CCs from animals primed with FSH (Sugimura *et al*., 2017). In addition, CC expression of transcripts associated with follicle atresia, including TSP1 downregulation and TGFB and TP53 upregulation, (Thomas *et al*., 2008), is suppressed by FSH-priming. FSH-priming also upregulated cumulus expression of CYP19A1, an indicator of healthy large follicles (Irving-Rodgers *et al*., 2009; Hatzirodos *et al*., 2014b). In the natural oestrus cycle of mono-ovulatory animals, a dominant follicle is ultimately selected for ovulation, whilst other growing follicles undergo atresia induced by granulosa cell signalling; as a result of the decrease in survival factors, predominantly FSH (Scaramuzzi *et al*., 2011). Hence, most COCs collected from antral follicles from a natural oestrus cycle ovary come from follicles at varying stages of atresia. Artificial control of follicular development through the administration of exogenous FSH rescues many antral follicles from atresia via promotion of survival by promoting anti-inflammatory mechanisms such as IRF7 (Sugimura *et al*., 2017). Whilst LH-induced ovulation induces an inflammatory cascade across the follicle, prior to the LH-surge, FSH is responsible for maintaining an anti-inflammatory state to maintain CC integrity. FSH causes suppression of STAT3 signaling (Ilha *et al*., 2015), and genetic perturbation of STAT3 expression has been shown to increase expression of IFNα/β response genes, including OAS and IRF7 (Wang *et al*., 2011). Hence, the net effect on COCs of FSH-priming of animals is decreased inflammatory signals and atresia, contributing to increased oocyte developmental competence (Fig. 2).

## Conclusion

An important function of follicular somatic cells in follicles approaching ovulation is to prepare the oocyte for final maturation, in part by promotion of cumulus EGFR signaling, as well as prevention of inflammation and promotion of cell-to-cell communication. The latter facilitates the efficient transfer of molecules from the somatic compartment of the follicle to the oocyte. These processes are likely to constitute important components of oocyte developmental competence in humans and ruminants.
